# Three dimensional mathematical modeling of violin plate surfaces: An approach based on an ensemble of contour lines

**DOI:** 10.1371/journal.pone.0171167

**Published:** 2017-02-06

**Authors:** Steven Piantadosi

**Affiliations:** Cedars-Sinai Medical Center, Los Angeles, California, United States of America; Fred Hutchinson Cancer Research Center, UNITED STATES

## Abstract

This paper presents an approach to describing the three dimensional shape of a violin plate in mathematical form. The shape description begins with standard contour lines and ends with an equation for a surface in three dimensional space. The traditional specification of cross sectional arching is unnecessary. Advantages of this approach are that it employs simple and universal description of plate geometry and yields a complete, smoothed, precise mathematical equation of the plate that is suitable for modern three dimensional production. It is quite general and suitable for both exterior and interior plate surfaces, yielding the ability to control thicknesses along with shape. This method can produce mathematical descriptions with tolerances easily less than 0.001 millimeters suitable for modern computerized numerical control carving and hand finishing.

## 1 Introduction

This paper describes an approach for constructing three dimensional mathematical models for the shape of violin plates that has not previously been used in the violin community. The method consists of two modeling steps beginning with ordinary plate contour lines. First, each contour is individually modeled with a general flexible equation independent of its elevation. Second, the coefficients from contour equations are quantitatively related to elevation using a second set of simple models. These steps jointly smooth and synthesize contour lines into a complete surface. The result also allows any number of additional contours to be drawn consistent with the originals and resulting surface. This represents essentially a full mathematical description of the plate surface shape that can be applied to both exterior and interior surfaces.

Models based on contours are useful in planning and executing three dimensional (3D) construction, and have been applied in cardiac imaging [1, 2]. They appear especially useful for computerized numerical control (CNC) methods that have become more affordable and commonplace in recent years for violin construction. Mathematical models of the physical structure of violins and the ability to alter them systematically may also contribute to understanding of acoustics. There is also a strong sense of aesthetics associated with mathematical approaches, although it is quite different from the visual, acoustic and traditional aesthetics surrounding musical instruments.

A variety of methods for constructing violin plates are in use, including visual and tactile guides, arching models, complex geometric prescriptions for constructing an outline and *f*-holes, and mathematical descriptors of cross sectional curves. Some traditional methods using those techniques are discussed in Roy [3] and Heron-Allen [4]. One cannot underestimate the ability of human training, memorization of the shape with eyesight, feel, and step-wise systematic production to produce a consistent and beautiful result using classical means. For construction via computerized methods, three dimensional scanning and reverse engineering have been applied. As successful as they can be, these methods are unable alone to produce a complete detailed quantitative model of the required shape, or more importantly, to yield methods for slight adjustments and modifications of the design under exact control. That capability would allow one to produce precise differences in output when carved under computer control.

To obtain high precision, we require a model, function, or equation, which I will denote by *z* = *Z*(*x*, *y*), that gives the correct surface elevation, *z*, for every *x* and *y* coordinate inside the outline of the plate. We can take the origin (*x* = 0, *y* = 0, *z* = 0) to be the centroid of the bounding box for the belly plate. However, the approach presented here is independent of the exact location of the origin. Knowing *Z*(*x*, *y*) permits exact specification of the surface, contours, cross sections or any other features of interest. This allows fine control over computerized tool paths or thicknesses.

A variety of idealizations with varying degrees of mathematical rigor have been applied to violin design. For example, classical arching has been said to follow the shape of a curtate cycloid [5] and various tools are available to make it so. The cycloid was first described by Galileo in 1599; a comprehensive discussion of its geometry was given by Proctor [6]. It is the path followed by a fixed point at radius *b* < *a*, where *a* is the radius of a rolling circle. The parametric equations for a curtate cycloid are *x* = *at* − *b Sin*(*t*) and *y* = *a* − *b Cos*(*t*). The curtate cycloid is probably not the correct literal shape for arching [7, 8], but may be useful because it represents a drawing tool/technique that can approximate the intended shape.

Muratov [9] suggests that the arching results when gravity acts on a flexible surface, and shows how it might be constructed using an outline form, cloth, and repeated coats of plaster. Photographs on his web site are not so convincing as to the accuracy of this method. This idea of gravitational action on a flexible sheet has an intuitive appeal, and an exact mathematical representation related to the catenary. The one dimensional catenary curve is well known, and the problem describing it lies at the origins of the calculus of variations [10]. A catenary surface however is a difficult mathematical beast [11]. The principles related to its specification were known to Lagrange [12], and the relevant nonlinear second order partial differential equation characterizing a catenary surface was known to Poisson [13]. It is

$$g\epsilon +\frac{c-g\epsilon z}{k^{2}}\left[\left(1+q^{2}\right)\frac{\partial^{2}u}{\partial x^{2}}-2pq\frac{\partial^{2}u}{\partial x\partial y}+\left(1+p^{2}\right)\frac{\partial^{2}u}{\partial y^{2}}\right]=0,$$ (1)

where $p=\frac{\partial u}{\partial x}$, $q=\frac{\partial u}{\partial y}$, $k=\sqrt{1+p^{2}+q^{2}}$, *g* is the gravitational constant, and *ϵ* is the density of the surface [11]. At the boundary, *u* = 0. This describes the equilibrium condition between the forces of gravity and tension in the fabric that is implied in Muratov’s construct.

**Fig 1:**
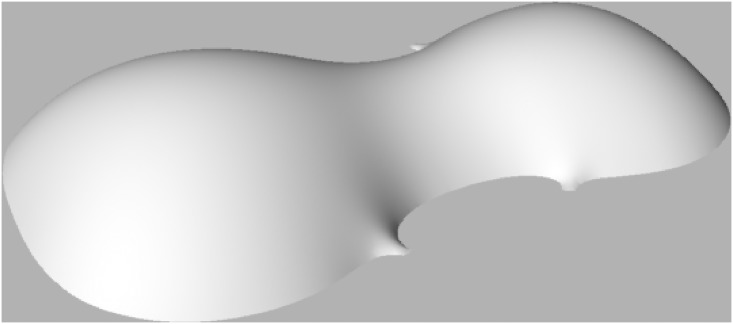
Catenary surface for violin outline based on numerical simulation of Eq 1. The *z* direction is exaggerated and reversed to demonstrate the non-monotonic surface that always results when tension is equal everywhere in the surface.

The solution to Eq 1 presents many challenges and is not easily available even in powerful computing packages, either analytically or numerically. However the solution can be simulated in the computer design package and physics engine Kangaroo [14] for Rhino/Grasshopper [15], and the results are disappointing for plate shapes. With the outline of a violin plate perimeter, a uniform tension two dimensional catenary assumes arching that is deeper in the upper and lower bouts than in the C bouts (Fig 1). It is important to note that Fig 1 has an expanded and arbitrary *z*-axis to demonstrate the shape. Hence even flatter surfaces also have a saddle that results from the requirement for uniform tension in Eq 1 and the narrow midsection. A uni-modal surface might be produced by varying the tension or altering the mass or force applied at each point or in various regions of the surface. This is likely to be a complex problem that may not have a simple physical analogy or easy mathematical representation.

Cycloids and catenaries evidently do not represent exact mathematical design prescriptions, although it is possible that clever modifications of them could find some future practical use. In this paper we take a more empirical approach to describing surface shape rather than prescribing it. Our starting point is with the contour lines that are commonly used to describe the shape of plates.

## 2 Methods

### 2.1 Modeling individual contours

Contour lines are a standard representation of three dimensional data like topographic maps. They have been used for centuries to describe the shape of violin plates because they are simple to measure and represent in two dimensions in charts or books. However, contours do not seem to have been taken seriously by those addressing a quantitative three dimensional model of the entire plate. The method described here begins with these contours, mathematically modeling each one individually, and then combining the individual models into a cohesive whole. Imperfections within or between contours can be corrected during the process.

I will illustrate this method using contour lines for an instrument following Sacconi [16] and modified personally. These contour lines in the form of an image file were imported into a computer aided design (CAD) package where they were traced by hand, induced to be symmetric about the midline (*Y*) axis, and converted to three dimensional coordinates. Direct digitization using raster to vector conversion software such as WinTopo [17] is an alternative. The curves were then imported into a CAD program and graphically placed at correct vertical heights to yield a skeletal 3D representation of a plate surface. Plate cross sections were not used in any way. For each plate, both exterior and interior surfaces were represented in this way. Each surface comprised 9 contour lines. Only the contours representing the smooth arching away from the plate edges were used, avoiding the problems associated with the sinking or re-curve, and corners on contours near the edges. Although the contours originated in a classical drawing, my personal changes, imperfections, and errors yield something that may not be a true copy of any particular instrument. This is irrelevant for the results, because the method can apply to any set of contours. The contours used are provided in a space delimited plain text file as described in Section 4. The details that follow are for the exterior back surface of the violin.

This simple skeletal representation can be used to reconstruct plates for CNC milling, for example using a 3D spline interpolation to fill in the arching between contours. This process brings out the need for smoothing, consistency, and adjustment of the contours. Even clean contour lines have slight internal inconsistencies, more so among each other than within a single contour. The resulting surface usually has creases or other irregularities after 3D spline reconstruction. While these anomalies can be smoothed in hand finishing, doing so might take one farther away from the intended surface. It is desirable to make the contour curves as consistent as possible with each other to minimize the need to remove imperfections. Perfectly consistent contour lines would not need *Z* smoothing.

The first step is to construct a basic model for a single contour. Each contour curve was converted to a set of points by sampling every 0.25 to 1.0 millimeters depending on its length. This yielded between about 100 to 1500 points per contour. The exact number of points sampled is not critical provided the shape is captured. Each contour curve is an oval or a pinched oval, suggesting a trigonometric base model as one would typically use for periodic data. Any single curve can be modeled very nicely as the set of points [*X*(*t*), *Y*(*t*)] with coordinates equal to the weighted sums,

$$X\left(t\right)=x_{0}+\sum_{k=1}^{m}p_{k}Sin\left(kt\right)$$ (2)

and

$$Y\left(t\right)=y_{0}+\sum_{k=1}^{n}q_{k}Cos\left(kt\right),$$ (3)

where the constants *p*_1_ … *p*_*m*_ and *q*_1_ … *q*_*n*_ are unique to each contour curve, and 0 ≤ *t* ≤ 2*π* (radians). This is formally called a “parametric” model because the coordinates are specified in terms of the dummy parameter *t*. The term *y*_0_ is present because the curves may not be vertically perfectly centered. An *x*_0_ term is expected to be negligible because the contours are explicitly horizontally centered and made symmetric around the *y* axis. The *z* coordinate is the same for all points in a given contour, by definition.

This model may seem a bit mysterious but will be more intuitive if one imagines polar coordinates. In Eqs 2 and 3, the dummy parameter *t* represents angular measure. The parametric equation for a circle is [*Sin*(*t*), *Cos*(*t*)] with 0 ≤ *t* ≤ 2*π*. Recall the trigonometric identity *Sin*^2^ + *Cos*^2^ = 1: hence the points on the circle are a constant distance from the origin. For points on an oval, the distance from the center varies with angle in a sinusoidal fashion. In model fitting with discrete points it is more convenient to replace angular measure with fractional curve length for each point, *f*_*i*_, which is easily calculated from the total number of sample points *n* as, *f*_*i*_ = *i*/*n*. For a circle, these would be identical to angular measure, but for an oval, fractional curve length only approximates angular measure. Also, in a pinched oval, angular measure is not monotonically increasing as the curve is traversed, whereas fractional curve length is monotonic.

Fitting this and other models to data points derived from contour curves, diagnostics, and graphics requires sophisticated mathematical modeling software. All the computations and graphics described herein were accomplished using Mathematica [18]. This report contains refined descriptions that might well be accomplished in simpler packages. However, development and exploration of these methods has required extensive capabilities, the details of which are not described here. A pdf file of the Mathematica program to accomplish all the calculations, tables, and graphics in this paper is available from the author.

**Fig 2:**
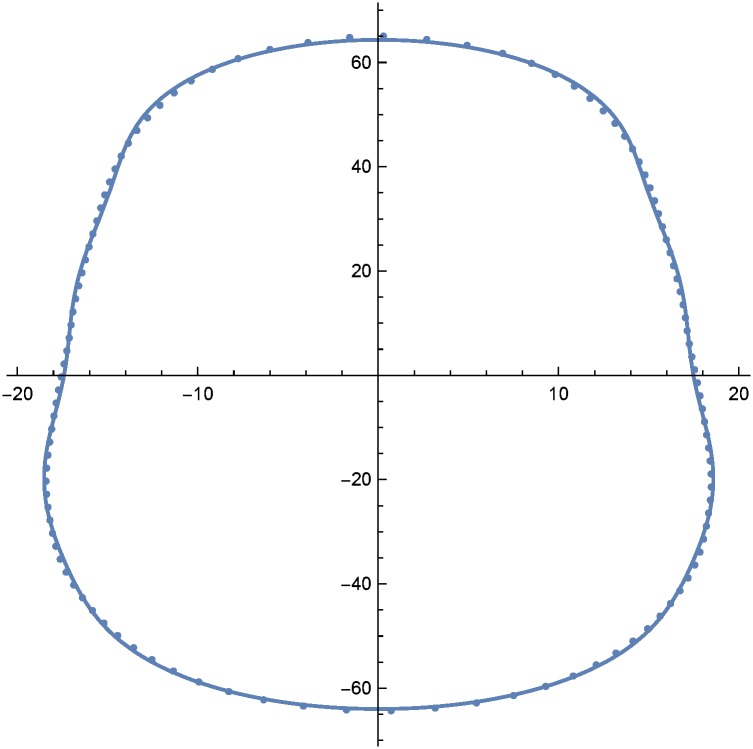
Model fit to a single contour at *z* = 13.26. Coordinates are in millimeters. The solid line represents the model, and points are derived from the actual contour line. Every fifth data point is shown. Very minimal lack of fit is evident in the most acute curvatures. The aspect ratio of this figure is 1.

For simple shapes like the pinched ovals of plate contours, the number of sine and cosine terms in the sums above can be relatively small. In all cases, I set *m* = *n* = 7 which is adequate for accurate matching of the model to all the contour lines. Inaccuracies are evident when the number of terms drops below 4 or 5. Using the method of least squares to fit Eqs 2 and 3 to the sampled points, the *p* and *q* coefficients or parameters that yield the closest overall fit of the model can be determined. These are presented in Tables 1 and 2 There, estimates for *x*_0_ and *y*_0_, which are generally close to zero have not been listed. An example contour and model fit is shown in Fig 2. The set of 7 *p* and 7 *q* coefficients (parameter estimates) for that fit are listed as contour (row) 4 of Tables 1 and 2, described below. Coefficients for a given contour curve are critical to shape, but at the same time are somewhat arbitrary. They are simply shape descriptors of the contour. What is not so obvious is that these shape descriptors demonstrate useful simple relationships to the *z* coordinate or elevation across the collection of contours as illustrated in the next section.

**Table 1:** Estimated shape coefficients for the parameters in Eq 2. Estimates of *x*_0_ are not listed.

Contour	Elevation	*p*_1_	*p*_2_	*p*_3_	*p*_4_	*p*_5_	*p*_6_	*p*_7_
1	14.8	1.991	−0.032	−0.018	−0.013	−0.010	−0.008	−0.007
2	14.6	6.512	−0.250	1.343	−0.008	0.490	0.012	0.177
3	14.1	13.779	−0.624	2.767	0.138	0.873	0.094	0.280
4	13.3	20.633	−1.146	3.794	0.146	1.119	0.077	0.489
5	12.1	28.886	−2.154	6.279	0.089	1.474	0.205	0.568
6	10.0	39.399	−4.644	10.625	0.144	1.965	0.624	0.340
7	8.0	50.359	−5.568	15.172	1.482	1.773	0.615	−0.135
8	5.8	64.021	−7.728	20.435	3.097	0.305	0.380	−0.514
9	4.6	76.464	−8.325	24.328	4.444	−1.603	−0.102	−0.768

**Table 2:** Estimated shape coefficients for the parameters in Eq 3. Estimates of *y*_0_ are not listed.

Contour	Elevation	*q*_1_	*q*_2_	*q*_3_	*q*_4_	*q*_5_	*q*_6_	*q*_7_
1	14.8	2.015	−0.016	−0.006	−0.003	−0.002	−0.001	−0.001
2	14.6	20.721	0.037	2.034	0.035	0.590	0.025	0.231
3	14.1	39.172	0.033	3.642	0.007	0.945	−0.031	0.311
4	13.3	56.795	0.199	5.350	0.157	1.469	0.091	0.547
5	12.1	77.406	0.485	6.984	0.365	1.702	0.249	0.528
6	10.0	100.034	1.679	8.000	1.146	1.273	0.805	0.036
7	8.0	120.333	1.873	8.663	1.025	0.756	0.724	−0.388
8	5.8	139.610	2.724	8.856	1.206	−0.075	0.907	−0.861
9	4.6	152.546	3.393	8.567	1.324	−0.888	1.015	−1.207

Contour models constructed in this way tend to be over-parameterized, meaning that a model with fewer parameters would fit nearly as well. The models can be made more parsimonious arbitrarily by using fewer coefficients. Another approach is to include a penalty term for the total number of parameters or the sum of their absolute values in the sum of squares equation. This will force “unnecessary” parameters toward zero values. The impact of excess terms can be that some parameters are imprecisely estimated. In many scientific applications, this would be unacceptable because inferential importance is often attached to the estimated values in models. This is not an issue here—we care only that the model represents the correct shape even if it is internally partially redundant. Too few parameters yield inaccurate fits to the contour, which disrupts the shape. So it is better to err on the side of slight redundancy.

Some may notice a similarity between typical contour curves and Cassini ovals, a family of quartic curves [19]. I will briefly endeavor to keep readers from wasting their time—these are not Cassini ovals, first investigated by the Italian astronomer Giovanni Domenico Cassini (1625–1712) in 1680. Cassini ovals are sections of a torus and are not flexible enough for the contours required in violin plate surfaces. The model I have proposed above is purely empirical, and is essentially a truncated Fourier series [20, 21].

### 2.2 Modeling coefficients versus elevation

**Fig 3:**
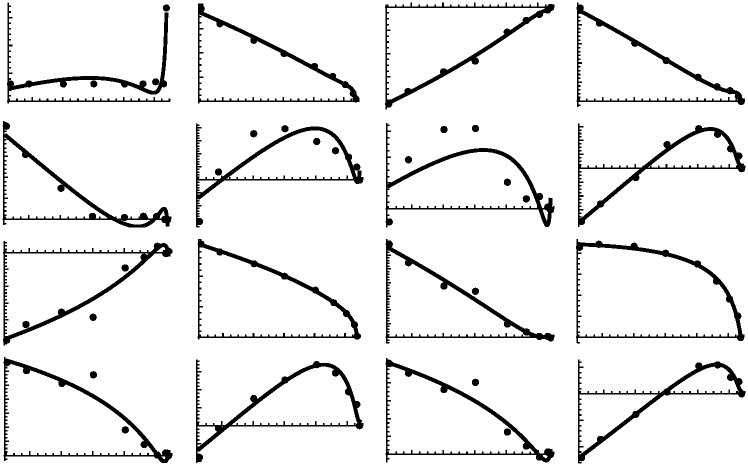
Relationships between elevation (horizontal axes) and estimated model coefficients (vertical axes). Plotted points are values from Tables 1 and 2. Solid lines represent interpolations as discussed in the text. The first eight plots are for estimates *x*_0_, *p*_1_–*p*_7_; the second eight plots are for *y*_0_, *q*_1_–*q*_7_. Note the horizontal axis is the same for each plot; the vertical axis is different for each plot.

To illustrate the relationship between the elevation of a contour and its shape characterizations, we can examine the set of coefficients for all 9 contour curves for the violin back exterior in Tables 1 and 2. In those tables, each row is a contour and each column is an estimated parameter. Note the fairly smooth pairwise relationships between elevation and the parameters, also shown in Fig 3. This suggests that each coefficient can be made a continuous function of elevation through numerical interpolation of those relationships. Each coefficient then becomes a formal function of elevation, denoted by *p*_*i*_(*z*) and *q*_*i*_(*z*) for *i* = 1 … 7 and elevation *z*. Using those interpolation functions, a contour can be constructed for any height, rather than only for the small set of elevations actually measured. Continuity of shape coefficients across elevation can be visualized in Fig 3 where each panel plots height versus one of the *p* or *q* coefficients. Construction of the solid lines in Fig 3 will be described below.

This step of the overall modeling process affords the greatest flexibility. Although we sought essentially a perfect fit for each contour curve, a perfect interpolation of shape coefficients across elevation is not required. For example, there seems to be some noise in the estimated values, perhaps due to over parameterization, asymmetry, or measurement error. Interpolations of contour coefficients can smooth the surface shape. Likewise, varying the interpolations will create differences in surface geometry. A common type of interpolation with cubic splines goes exactly through the coefficient-height pairs and yields smooth minimalist curves between the data points. The resulting surface will contain the original contours exactly.

In Fig 3 we can see the relationship between elevation and shape coefficients. In each plot, the points are the fitted contour coefficients and the line drawn is a smoothed continuous relationship based on the cubic polynomial model

$$p_{i}=a+b\log\left(W-z\right)+c\log{\left(W-z\right)}^{2}+d\log{\left(W-z\right)}^{3}$$ (4)

where *a*, *b*, *c*, and *d* are new parameters to be estimated and *W* is a fixed constant taken to be slightly larger than the largest *z*. This model is cubic overall rather than cubic between points as a spline would be. As such it yields large scale smoothing rather than small scale smoothing, and was chosen for simplicity and empirically good fit. A model without logarithms could also work—the exact form of this interpolation is essentially irrelevant except that it reasonably represents the *p*_*i*_. The estimates for each *p* and *q* coefficient are shown in Table 3. There is one equation for each coefficient, but the set of *a*, *b*, *c*, *d*’s is immaterial except to determine one shape coefficient for given *z*. The interpolations do a reasonable job of representing the estimated coefficients.

**Table 3:** Estimated parameters from Eq 4 that make shape coefficients functions of elevation. For all these regressions, *W* = 17.

Coefficient	*a*	*b*	*c*	*d*
*p*_1_(*z*)	−77.533	161.479	−95.539	21.222
*p*_2_(*z*)	−1.507	4.435	−3.636	0.327
*p*_3_(*z*)	−13.396	28.794	−18.552	5.171
*p*_4_(*z*)	−7.251	17.679	−13.541	3.316
*p*_5_(*z*)	6.690	−17.586	14.944	−3.653
*p*_6_(*z*)	3.447	−8.220	6.031	−1.314
*p*_7_(*z*)	1.650	−2.693	−0.662	−0.107
*q*_1_(*z*)	−164.517	316.284	−150.451	29.709
*q*_2_(*z*)	0.965	−2.187	1.233	−0.002
*q*_3_(*z*)	−14.466	25.896	−9.805	1.264
*q*_4_(*z*)	2.303	−5.568	4.030	−0.788
*q*_5_(*z*)	−3.563	5.609	-0.886	−0.357
*q*_6_(*z*)	1.645	−3.923	2.776	−0.527
*q*_7_(*z*)	−2.505	4.682	−1.973	0.128

Why is this useful? Whereas each contour model is restricted to a fixed elevation, these new models smooth and connect the shape coefficients across heights—*z* is not restricted to fixed values within the range for the plate. In other words, we can draw a contour at any elevation, *z*, by calculating the needed shape coefficients from the appropriate Eq 4 and plugging the coefficients in Eqs 2 and 3. We have replaced a set of discrete *p*_*i*_ and *q*_*i*_ by functions *p*_*i*_(*z*) and *q*_*i*_(*z*). The shape coefficients change smoothly across heights because the slope or derivative of Eq 4 changes smoothly. The resulting contours are jointly consistent with the originals used to construct the models and connect them ideally.

**Fig 4:**
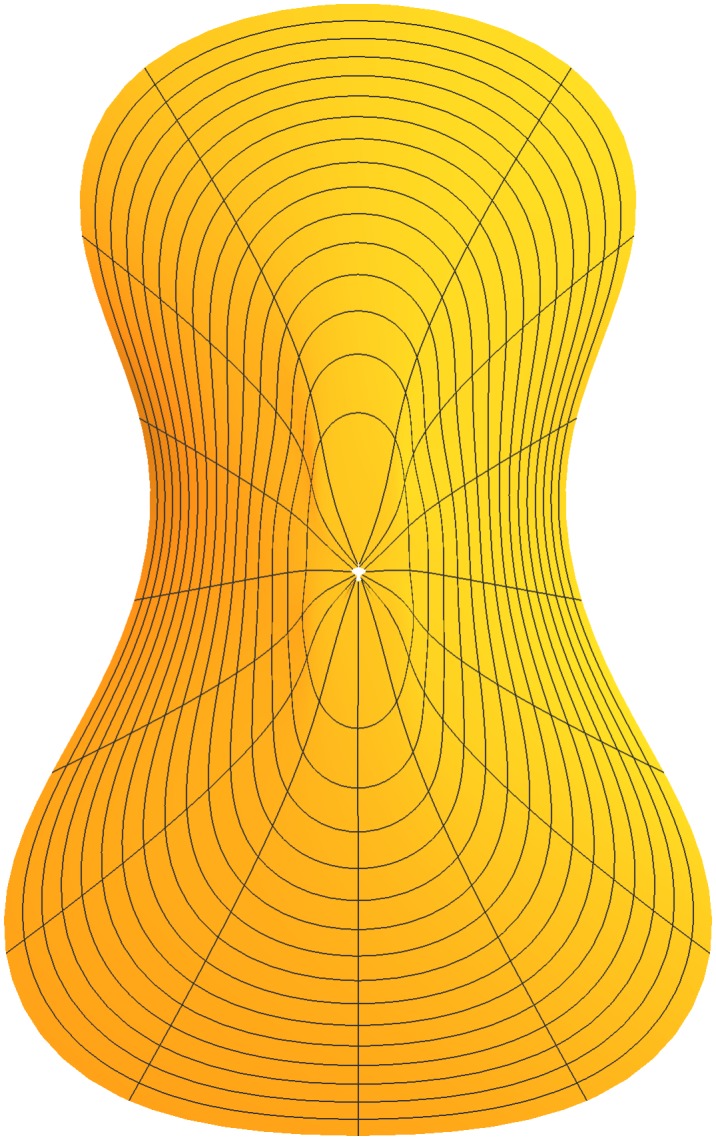
Surface plot derived from smoothed contours as described in the text.

Additionally, we have replaced 14 × 9 = 126 coefficients (ignoring *x*_0_ and *y*_0_) for a given set of contours with 14 × 4 = 56 parameters that smoothly determine a contour at any elevation. This represents a terrific advantage in efficiency. We are now in a position to see a smoothed version of a violin plate with any contours of our choosing. An example is shown in Fig 4 which shows the exterior back plate. In that figure, space between the contour lines is interpolated to illustrate the smoothness of the surface. Such interpolation is not strictly necessary because we are free to calculate dozens or hundreds of contour lines to fill in the gaps, although this would be hard to draw. In any case, modeling allows or requires the contour shape coefficients to change smoothly with plate elevation. This induces the contours to change gently with elevation, which creates smoothness in the surface. In addition we have a way to characterize every point on the surface mathematically.

There is nothing special about Eq 4 as a representation of the way a shape coefficient changes with elevation. A number of simple models with continuous first and second derivatives might reasonably represent the data in Fig 3. This contrasts with the model in Eqs 2 and 3, which are fundamentally connected to the periodicity in individual contours. Alternatives to Eq 4 will yield slightly different relationships between coefficients and elevation, which will in turn alter the shape of the surface. The resulting differences are likely to be small, but there is no reason not to explore them. What should be avoided is simply connecting the coefficients with line segments. That strategy would not yield continuous derivatives upon which smoothness depends and likely result in creases in the surface.

**Fig 5:**
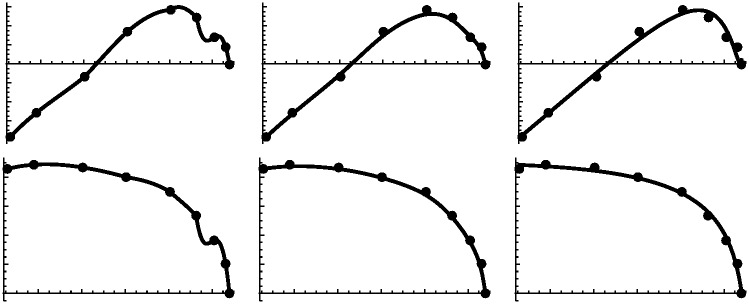
Examples of three methods of interpolating elevation versus coefficient points. From left to right they are cubic spline, b-spline, and cubic polynomial. The top row is for coefficient *q*_1_ and the bottom row for *p*_5_ as examples. All methods are smooth on a fine scale. The b-spline and cubic polynomial are also smooth on a large scale but may not fit the data points exactly.

**Fig 6:**
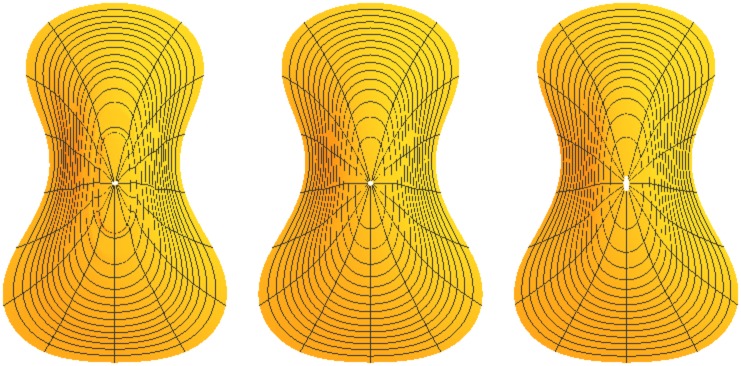
Surface plate shapes resulting from three interpolation strategies. From left to right they are based on cubic spline, b-spline, and cubic polynomial interpolation. Note that the radial lines are not true radii due to the fact that fractional arc length rather than angular measure was used in the contour model as discussed in Section 2.1.

As examples, two additional interpolation strategies were applied to the shape coefficients. One is an ordinary cubic spline [22, 23] with knots set at the observed coefficient-height pairs. This interpolation reflects the exact values at the knots, and connects them with piecewise smooth cubic polynomials. The overall interpolations can appear irregular, but the resulting surface will be smooth. The third interpolation strategy is based on b-splines [22, 23], which use the coefficient-height pairs as control points for the spline. The resulting curve does not necessarily pass exactly through the coefficient-height points, but is responsive to their influence. The b-spline interpolation shows an intermediate degree of smoothness between the overall cubic polynomial and the piecewise spline. The interpolation representations of coefficient-height pairs is shown in Fig 5. Surfaces that result from these three interpolation strategies are shown in Fig 6.

## 3 Results

### 3.1 The surface equation

In the beginning of this paper I indicated that it would be desirable to have the function *z* = *Z*(*x*, *y*) that determines the elevation, *z*, of the plate for any *x*-*y* coordinates inside the plate boundary. There are at least two ways to accomplish this. The first is based on the fact that any point (*x*, *y*) can be projected vertically to intersect a contour line. Finding the contour that contains the given (*x*, *y*) explicitly yields the elevation *z*. To see how this can be done, rewrite Eq 2 in the form

$$X\left(z,t\right)=\sum_{k=1}^{m}p_{k}\left(z\right)\;Sin\left(kt\right),$$ (5)

to indicate the effect of elevation by virtue of the *p*_*i*_(*z*) regression fits in Fig 2 and Table 3. Similarly for Eq 3 and *q*_*i*_(*z*). We can then numerically solve the two simultaneous equations

X(z,t)=x, (6)

Y(z,t)=y (7)

for any given *x* and *y* for the two unknowns *z* and *t*. Focus will be on the solution for *z*. The value for the dummy parameter *t* is of no interest, but it is necessary to find a solution for the system. Various methods could be used to solve this system, but one that works reasonably well is to numerically minimize the quantity

$$T={\left(x-\sum_{k=1}^{m}p_{k}\left(z\right)\;Sin\left(kt\right)\right)}^{2}+{\left(y-\sum_{k=1}^{m}q_{k}\left(z\right)\;Cos\left(kt\right)\right)}^{2}$$ (8)

with respect to *z* and *t*. A few examples are shown in Table 4.

**Table 4:** Some example values for *Z*(*x*, *y*) from Eq 8.

*x*	*y*	*Z*(*x*, *y*)
55	-75	7.5
-27	43	15.6
15	30	15.3
-45	-45	8.2

Although correct in theory, this method for *Z*(*x*, *y*) is somewhat inconvenient. Generating a set of (*x*, *y*, *z*) coordinates is relatively slow and subject to errors because of the computational burden. If (*x*, *y*) lies outside the contour boundary, the some solution methods may yield nonsensical values because there is no basis to extrapolate there. The dummy parameter, *t*, which was a mere convenience in Eqs 2 and 3 is now integral to the solution of Eq 8. But as illustrated in Fig 6, the radial lines are irregular as a consequence of using fractional arc length rather than angular measure for *t* in Eqs 2 and 3 (discussed in Section 2.1).

An alternative is to create a 3D interpolating function across a new family of smooth contours. Many contours can be generated to give the interpolation extensive support. The interpolating function object, which I will denote by *Z*_*h*_(*x*, *y*), passes exactly though the contours because they are smooth across elevation, and yields the *z* coordinate of any point (*x*, *y*). The figures above are filled in using exactly such a 3D interpolation function. *Z*_*h*_(*x*, *y*) is also a small computational burden because it represents the entire surface rather than a single (*x*, *y*, *z*) point. But it is probably the fastest method to obtain coordinates for the surface. Here again interpolations cannot be extended beyond the outer contour.

However we accomplish it, assume that we have a set of (*x*, *y*, *z*) coordinates representing the surface of our violin plate. Those points should be of sufficient density that graphics or simple interpolations in standard CAD/CAM packages will represent the surface accurately for further processing. An advantage of surface modeling compared to solid modeling is that partial control over plate thickness can be maintained by having separate interior and exterior surfaces that can be machined independently. Alternatively one could assemble surfaces into solid objects in CAD/CAM software for machining. It is within the scope of inexpensive software to generate raster tool paths from such solids. These will be necessarily a little coarse but acceptable because hand finishing, such as scraping, is required. There are much better tool paths than those based on rasters for these plates, but that is altogether a different complex topic.

### 3.2 Results of modeling four plate surfaces

**Fig 7:**
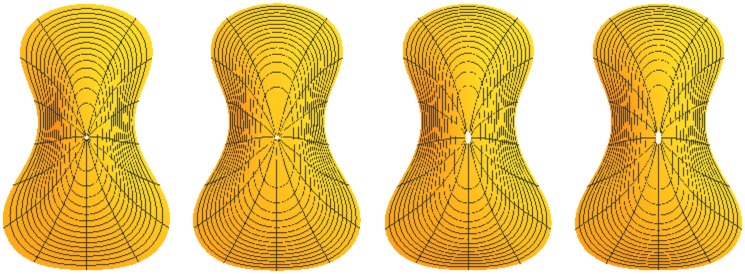
Results of mathematical surface modeling. From left to right the figures are belly exterior, belly interior, back exterior, and back interior.

The methods described above were applied to both inside and outside surfaces of belly and back plates (four surfaces total). The interior contours of each plate were derived by me from the exterior contours and approximate plate thicknesses provided by public and personal sources. Additional thickness is to be removed with plate tuning. This permits near final CNC carving with final tuning by hand. Results of surface modeling are shown in Fig 7.

My emphasis in this report is on the method rather than the output. All of the mathematics employed is purely descriptive rather than prescriptive. Provided reasonable choices are made where the methods are flexible, the final surface geometry will contain the initiating contour curves up to the precision of the oval model, which is quite high, and the tolerance of the coefficient interpolations, which can be more relaxed. With testing, revision, and a smarter tool path than simple raster, it is possible to produce a machined plate of near final dimension. It should also be evident that these methods can produce plates for instruments larger than the violin, although I have personally not done so.

## 4 Discussion

The goal of this work has been to provide a precise mathematical characterization of the surface of a violin plate. My motivations are oriented toward labor saving technologies, CNC carving in particular. Having control over details of the process, even when leaving significant hand finishing for aesthetics and acoustics is essential. Beginning such an effort from common simple empirical characterizations of shape such as contour curves is highly desirable, as opposed to assuming a specific mathematical form *a priori*. I have avoided some specific formal mathematical prescriptions because they seem not to be correct, but more importantly because they are unnecessary.

It is my hope also to illustrate that descriptive mathematics in service of this art can be as aesthetic, personal, and unique as hand work. It is possible that someone would adopt such methods, settle on a convenient design, and produce a string of very similar looking violins by CNC machine. This might be something to explore if one were studying varnish methods, but is hardly the goal here. Less nuanced methods would suffice for production on an impersonal scale. What seems more interesting is to refine a personal model or use this as a method for copying interesting historic pieces, and save some labor. Small changes precisely done could be assessed for their effect on acoustics for example. In such an endeavor, variability because of differences in wood could be reduced by replication. Without such replication, one could never be sure if a difference in sound is due to wood or nuances in construction.

Much has been made of mathematics possibly underlying classical instrument construction. These perspectives range from simple use of the golden ratio, to formal constructions of the outline or inside mold and corresponding landmarks, to prescriptions for arching, equations for spirals, and so on. Although I personally have a love for applied mathematics, I consider such attempts to be hollow and useless. The artists who contributed to this form over the centuries were not mathematicians, and there is no underlying theory to be discovered by such methods.

This is not to say that modern technology has no role to play in the art. For example we can employ tools that are more durable, sharper, or more precise than old ones. We can use modern chemistry, material science, and imaging to allow better understanding. We can use technology to save labor, assuming that labor is actually unnecessary for the greater goal. In some cases, the labor of hand work is quite pleasurable, part of the experience, and contributes to an essential feel for the object. In other cases, it is simply hard work, a distraction, and might be minimized by a better hand tool or a refined technique. The perspective is probably very personal.

In the present case, we can combine the simplest contour descriptors with sophisticated modeling to assist what is becoming a standard approach to plate carving, at least in the rough. The flexibility of this method is enormous, and the mathematics is applied as a servant rather than as a tyrant.

## Supporting information

S1 File This file contains x,y,z coordinates for the 9 contour lines used in the text. Coordinates are space delimited. Contours are distinguished by their z coordinates.
(TXT)
